# Chronic intrahypothalamic rather than subcutaneous liraglutide treatment reduces body weight gain and stimulates the melanocortin receptor system

**DOI:** 10.1038/ijo.2017.98

**Published:** 2017-05-16

**Authors:** K Kaineder, T Birngruber, G Rauter, B Obermüller, J Eichler, J Münzker, W Al-Zoughbi, S I Mautner, S S Torekov, B Hartmann, P Kotzbeck, T R Pieber

**Affiliations:** 1 Division of Endocrinology and Diabetology, Department of Internal Medicine, Medical University of Graz, Graz, Austria; 2 Joanneum Research, Health – Institute for Biomedicine and Health Sciences, Graz, Austria; 3 Division of Biomedical Research, Medical University of Graz, Graz, Austria; 4 Division of Biomedical Research, Alternative Biomodels and Preclinical Imaging, Medical University of Graz, Graz, Austria; 5 Institute of Pathology, Medical University of Graz, Graz, Austria; 6 Department of Biomedical Sciences and The NNF Center for Basic Metabolic Research, University of Copenhagen, Copenhagen, Denmark

## Abstract

**Background:**

The GLP-1 receptor agonist liraglutide is marketed for obesity treatment where it induces body weight reduction possibly via the hypothalamus, which regulates energy homeostasis. In animal studies, acute liraglutide treatment triggers satiety, weight loss and activates thermogenesis in adipose tissue. However, the precise mechanisms how liraglutide affects in particular chronic weight loss are still under investigation.

**Objectives:**

We aimed to evaluate whether chronic hypothalamic or chronic subcutaneous administration of liraglutide induces sustained weight loss through altered adipose tissue function and to what extent hypothalamic neuronal appetite regulators are involved in the liraglutide-induced weight loss in healthy lean rats on a normal diet.

**Materials/Methods:**

We continuously administered liraglutide either intrahypothalamically (10 μg per day) or subcutaneously (200 μg kg^−1^ per day) for 28 days to lean Sprague Dawley rats (*n*=8 each). We assessed changes in body weight, adipose tissue mass, adipocyte size and adipose tissue volume in the abdominal region by using micro-CT. We analyzed genetic expression patterns of browning, thermogenic and adipocyte differentiation regulators in adipose tissues as well as particular neuronal appetite regulators in the hypothalamus.

**Results:**

Intrahypothalamic liraglutide administration induced an 8% body weight reduction at day 9 compared with the control group (*P*<0.01) and a 7% body weight loss at day 9 compared with subcutaneous liraglutide treatment (*P*<0.01), supported by a significant reduction in adipose tissue mass and volume with intrahypothalamic liraglutide administration (*P*<0.05). Our data show that chronic intrahypothalamic liraglutide treatment triggered an 18-fold induction of the hypothalamic *mc4r* gene (*P*<0.01) accompanied by a significant increase in circulating thyroxine (T4) levels (*P*<0.05).

**Conclusions:**

Chronic intrahypothalamic liraglutide administration resulted in a profound reduction in body weight and fat mass loss most likely mediated by the hypothalamic melanocortin system rather than by adipose tissue browning or improved thermogenesis.

## Introduction

Liraglutide is a long-acting GLP-1 receptor (GLP-1R) agonist and has been FDA and EMA approved for the treatment of obesity.^1^ Liraglutide lowers blood glucose levels by stimulating insulin secretion and by inhibiting glucagon secretion in a glucose-dependent manner.^2^ In addition to its glucoregulatory effects, liraglutide reduces caloric intake and subsequently leads to sustained but moderate weight loss.^3^ The glucoregulatory mechanisms of liraglutide are well known, but the mediating mechanisms underlying the anorectic and body weight-reducing effects are less clear.^4^ Acute central liraglutide treatment (24 h, ventromedial hypothalamic nucleus) in rodents has been shown to result in body weight loss independent of caloric intake. Body weight loss was attributed to the stimulation of thermogenesis in the brown adipose tissue (BAT) and browning of white adipose tissue (WAT) via the hypothalamic AMP-activated protein kinase pathway.^5^ Peripheral liraglutide administration led to a reduced ability to suppress food intake and maintain body weight in diet-induced obese mice with a GLP-1 receptor knockdown in the central nervous system.^6^ In addition, peripheral liraglutide administration in rats with pharmacological antagonized GLP-1 receptors in the brain resulted in a reduced ability to suppress food intake.^7^ A recent study more specifically identified POMC/CART neurons in the arcuate nucleus of the hypothalamus as the main mediators of weight-reducing effects after chronic peripheral liraglutide treatment.^8^ But peripheral administration of liraglutide often leads to only limited effects mediated in the brain because only small amounts of the large liraglutide–albumin complex can cross the blood–brain barrier.^4^ In humans, such a limited passive transport of peripherally administered liraglutide to the cerebrospinal fluid did not correlate with the observed weight loss.^9^

Combining the existing data, acute pharmacological interventions do not necessarily reflect the physiological, chronic regulation of feeding by the brain. A direct comparison of chronic central and chronic peripheral liraglutide administration in a single study would help to link existing studies investigating either central or peripheral administration. Such comparative data on chronic central and peripheral effects of liraglutide are necessary to identify new pathways to improve the pharmacological benefits of chronic obesity treatment.

We aimed to evaluate whether chronic hypothalamic or chronic subcutaneous administration of liraglutide induces sustained weight loss through altered adipose tissue function and to what extent hypothalamic neuronal appetite regulators are involved in the liraglutide-induced weight loss.

## Materials and methods

### Animal models

Male Sprague Dawley rats (12–15 weeks old and weight matched; Charles River Laboratories, Sulzfeld, Germany) were housed under conditions of controlled temperature (23 °C) and a 12 h light/dark cycle. The rats had *ad libitum* access to water and standard laboratory diet. Immediately after the rats were killed by rapid decapitation, tissue samples (hypothalamus, brown and white adipose tissue) were removed, frozen in liquid nitrogen and kept at −80 °C until further analysis. All animal experiments were approved by the Austrian Federal Government (BMWF-66.010/0010-WF/V/3b/2015) and were performed in accordance with Directive 2010/63/EU on the protection of animals used for scientific purposes.

### Study design

The study design (non-blinded) was based on four groups (each *n*=8). Two groups received treatment (liraglutide) either intrahypothalamically (central) or subcutaneously (peripheral) and two groups received placebo (artificial cerebrospinal fluid (aCSF) or 0.9% NaCl) again either intrahypothalamically or subcutaneously. Group 1: intrahypothalamic liraglutide; group 2: intrahypothalamic vehicle (aCSF); group 3: subcutaneous liraglutide; group 4: subcutaneous vehicle (NaCl). Group 2 and group 4 (treated with placebo) served as control groups.

To determine the acute (24 h) effects of liraglutide on body weight, adipose tissue mass and gene expression patterns (hypothalamus, WAT and BAT), liraglutide (10 μg per animal) or aCSF (2 μl) were injected just above the dorsal part of the paraventricular nucleus (stereotactic coordinates: anteroposterior: 1.7 mm, mediolateral: 0.6 mm, dorsoventral: 7.6 mm). For chronic administration, the rats were continuously treated for 28 days with either 10 μg per day per animal intrahypothalamic liraglutide or with 200 μg kg^−1^ per day subcutaneous liraglutide via osmotic pumps. The administered dose of intrahypothalamic liraglutide was chosen on its ability to significantly inhibit feeding and induce body weight loss.^10, 11^ The dose of subcutaneous administration was chosen to differentiate between the effect of peripheral (subcutaneous) and central (intrahypothalamic) liraglutide.^8^

### Implantation of osmotic pumps

Before implantation, rats were individually placed in an anesthesia induction chamber (Rothacher, Heitenried, Switzerland) induced with 4 vol% isoflurane (Isoflo, Esteve Farma, Carnaxide, Portugal) in 100% oxygen with a delivery rate of 5 l min^−1^ until loss of righting reflex. The rats were anesthetized using 0.1 ml kg^−1^ of an injectable anesthetic (0.5 mg kg^−1^ midazolam, 5 μg kg^−1^ fentanyl, 5 mg kg^−1^ medetomidin; 1 ml per 1 kg body weight, ERWO Pharma GmbH, hameln pharma plus GmbH, Vienna, Austria). Anesthesia was maintained with isoflurane in 100% oxygen at a flow of 1.5 l min^−1^. For 28 days continuous drug administration, we chose the pump models 2ML4 (1997.2 μl fill volume, 2.55 μl h^−1^ flow rate) for subcutaneous and the model 2004 (234 μl fill volume, 0.22 μl h^−1^ flow rate) for intrahypothalamic administration (ALZET Durect, Cupertino, CA, USA). Osmotic pumps were implanted according to the manufacturer’s instructions by creating a pocket at the midscapular region using a hemostat and inserting the filled pump in the pocket.

### Implantation of intrahypothalamic cannula

The induction of anesthesia for stereotactic surgery was the same as for the implantation of osmotic pumps as described above. Stereotactic surgery and postsurgical pain control was performed as previously described.^12^ The brain cannula (PlasticsOne, Bilaney Consultants, Düsseldorf, Germany) was implanted just above the dorsal part of the paraventricular nucleus (stereotactic coordinates: anteroposterior: 1.7 mm, mediolateral: 0.6 mm, dorsoventral: 7.6 mm). For intrahypothalamic administration, the cannula was connected to the osmotic pump that was filled with liraglutide (1.91 μg h^−1^; Novo Nordisk, Bagsværd, Denmark) dissolved in aCSF (Harvard Apparatus, March-Hugstetten, Germany). The coordinates were histologically verified after injection of 1% Evans Blue dye and spectrophotometrically by analyzing sodium fluorescein dye in hypothalamic tissues (Supplementary Material).

### Assessment of body weight, adipose tissue mass and size

Body weight was continuously assessed on a precise laboratory scale (Competence CP3202S-0CE, Sartorius AG, Göttingen, Germany). After 28 days, the rats were killed and freshly excised adipose tissues depots (inguinal WAT, epididymal WAT, interscapular BAT) were weighed on an analytical balance (M-Power AZ214, Sartorius AG, Göttingen, Germany). Epididymal (eWAT) and inguinal (iWAT) white adipose tissue and interscapular brown adipose tissue (iBAT) were isolated, fixed in 4% paraformaldehyde overnight at room temperature and embedded in paraffin. The embedded tissues were cut in 5 μm-thick sections and stained with hematoxylin and eosin. The adipose tissue sections were evaluated using the Olympus BX51 microscope and representative areas from these sections were captured by using the Olympus camera 4A14690. The images were acquired with a standard × 20 microscope objective lens. The imaging area was selected according to a high prevalence of adipocytes with an intact cell membrane and minimal adjacent tissues such as blood vessels, muscle and inflammatory cells. Adipocytes with unclear cell borders were excluded from the analysis. Image annotation was performed manually by using the Olympus analySIS five software and the mean adipocyte size was calculated for each type of adipose tissue.

### Assessment of visceral and subcutaneous adipose tissue volume

Abdominal adipose tissue volumes of visceral and subcutaneous depots were measured with a micro-CT scanner (Siemens Inveon micro-CT, Siemens Healthcare GmbH, Erlangen, Germany; energy settings: 200 mA, 80 kV, 1200 ms) with Siemens Inveon Acquisition Workplace software (version 1.2.2.2) before (baseline) drug treatment and after 28 days of chronic infusion of liraglutide or vehicle. Data were reconstructed using filtered back projection and algorithm of Feldkamp in Siemens Inveon Acquisition workplace. The reconstructed data sets were exported to DICOM format using Siemens Inveon Research Workplace and post processed regarding adipose tissue volumes using Materialise MIMICS v.19 (Materialise, Leuven, Belgium). We focused on the abdominal region, as scanning the abdominal adipose depots provides sufficient information to estimate the total body fat and monitor site-specific changes in adiposity and reduces scanning time.^13^

### RNA isolation, complementary DNA transcription and reverse transcribed-quantitative PCR

QIAzol Lysis Reagent (QIAGEN GmbH, Hilden, Germany) was used for tissue lysis. The total RNA content was isolated from homogenized adipose and hypothalamic tissue by using the RNeasy Mini Kit (QIAGEN GmbH, Hilden, Germany) including on-column DNaseI treatment. RNA quantity was measured on NanoDrop (NanoDrop 2000c, ThermoFisherScientific GmbH, Vienna, Austria) and 1 μg total RNA was reverse transcribed to cDNA by using the iScript advanced cDNA synthesis kit (Bio-Rad Laboratories, Vienna, Austria). Gene expression analysis via quantitative PCR was performed using TaqMan Universal PCR Master Mix (Life Technologies, Carlsbad, CA, USA) or LightCycler 480 SYBR Green I Master Mix (Roche, Vienna, Austria) according to the manufacturer’s instructions on a Roche LightCycler 480 Instrument (Roche Austria, Vienna, Austria). The sequences of primers and probes are listed in the Supplementary Material.

### Measurement of metabolic and hormonal parameters

Plasma glucose levels were assessed by using the Accu-Check Performa system (Roche Diabetes Care Austria GmbH, Vienna, Austria). Non-esterified free fatty acids (NEFA) were measured via the enzymatic colorimetric NEFA-HR(2) assay kit (WAKO Diagnostics, Richmond, VA, USA). Plasma-free glycerol content was colorimetrically quantified using free glycerol reagent (Sigma Aldrich, Vienna, Austria). Plasma triglyceride concentrations were analyzed by using the Infinity Triglyceride Assay (ThermoScientific, Vienna, Austria). Circulating leptin, insulin, thyridiodine (T3) and thyroxine (T4) concentrations were measured with the Mesoscale Multiplex Array Elisa System (Mesoscale Diagnostics, Rockville, MD, USA). All the measurements were performed according to the manufacturer’s instructions.

### Statistical analysis

The data are expressed as mean±s.d. compared with the corresponding control or treatment group. The sample size was chosen based on published literature and to perform descriptive statistics. Shapiro–Wilk test was used to scrutinize normality. Either unpaired Student's two-tailed *t*-test or Mann–Whitney *U*-test were used to determine statistical significance of data comparing the two groups. Subsequently, the *P*-values were corrected for multiple testing using the Benjamini–Hochberg procedure. To determine equality of variances between and within subjects, Levene’s and Mauchley’s sphericity tests with Greenhouse–Geisser correction were used. For repeated body weight measurements, we used two-way repeated-measures mixed-model analysis of variance plus Bonferroni *post hoc* analysis. The corrected *P*-values <0.05 were considered statistically significant. The calculations were performed in GraphPad Prism Mac 5.0b software (La Jolla, CA, USA) and SPSS Statistics 23 (IBM, Ehningen, Germany). Relative gene expression levels (messenger RNA (mRNA)) were analyzed using the 2^(−ΔΔCt)^ method.^14^

## Results

### Central chronic liraglutide treatment reduces body weight gain and promotes adipose tissue mass reduction

**Figure 1:**
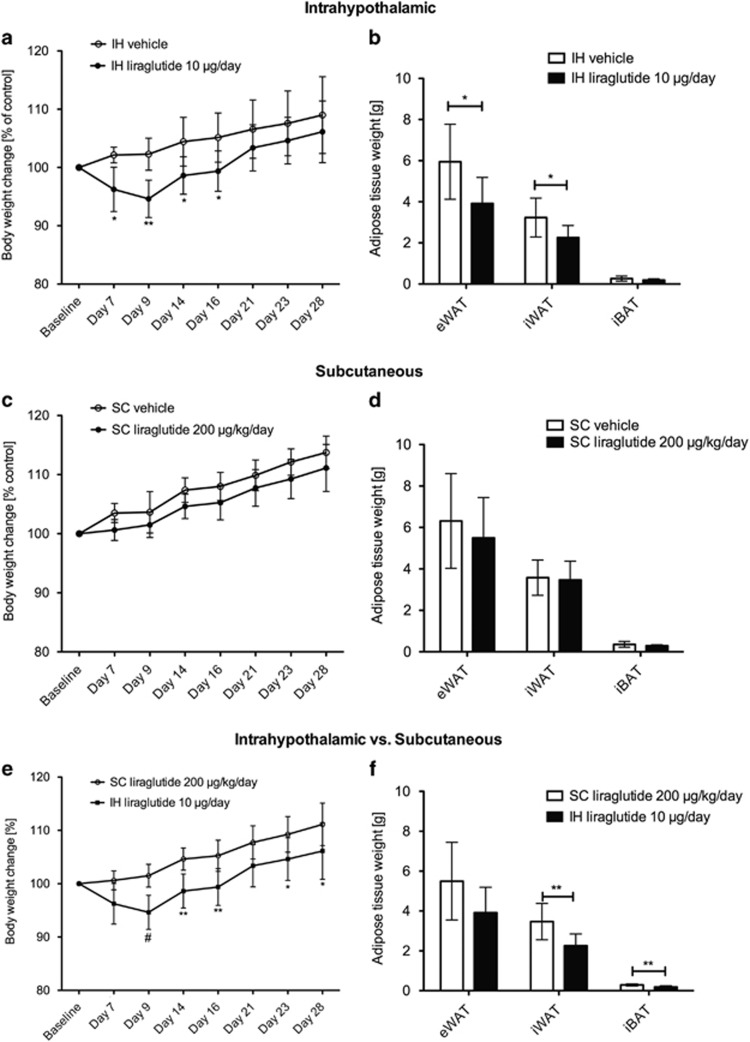
Central IH administration of liraglutide leads to reduced overall body weight gain and adipose tissue mass reduction. (**a**) Reduced body weight gain over 28 days triggered by continuous IH administration of liraglutide (IH 10 μg per day) compared with the corresponding control group (vehicle, aCSF) interaction *P*=0.0025, time *P*<0.0001, treatment *P*<0.0210. (**b**) Reduced adipose tissue weights from eWAT, iWAT and iBAT depots after 28 days of IH liraglutide administration. (**c**) No change in body weight from baseline to day 28 triggered by continuous SC administration of liraglutide (200 μg kg^−1^ per day) compared with corresponding control group (vehicle, 0.9% sNaCl) interaction *P*=0.3379, time *P*<0.0001, treatment *P*=0.0295. (**d**) No change in adipose tissue weights from eWAT, iWAT and iBAT depots after 28 days of SC liraglutide administration. (**e**) Reduced body weight over 28 days after chronic IH liraglutide treatment compared with SC liraglutide treatment. Interaction *P*=0.0010, time *P*<0.0001, treatment *P*=0.0024. (**f**) Reduced adipose tissue weights from eWAT, iWAT, iBAT after 28 days of IH liraglutide versus SC liraglutide administration. Data are given as mean±s.d. of seven to eight animals per group. (*P<0.05, **P<0.01, ^#^*P*<0.001). IH, intrahypothalamic; SC, subcutaneous.

Intrahypothalamic treatment resulted in a significant body weight loss of 4% at day 7 (*P*<0.05) and 5% at day 9 (*P*<0.05) compared with baseline. Compared with the control group (intrahypothalamic vehicle), chronic intrahypothalamic liraglutide treatment significantly reduced body weight gain by 6% at day 7 (*P*<0.05), 8% at day 9 (*P*<0.01), 6% at day 14 and day 16 (*P*<0.05; Figure 1a). A 3% reduction in body weight gain was maintained from day 21 to day 28 compared with intrahypothalamic control group (Figure 1a). Intrahypothalamic liraglutide-treated rats had significantly reduced epididymal and inguinal fat mass compared with the control group (*P*<0.05; Figure 1b). Chronic peripheral (subcutaneous) administration of liraglutide did neither affect body weight (Figure 1c), nor did it reduce adipose tissue mass (Figure 1d). Compared with the subcutaneous liraglutide treatment group, chronic intrahypothalamic liraglutide treatment induced a significant body weight loss at day 9 (*P*<0.001), at day 14 and 16 (*P*<0.01), at day 23 and 28 (*P*<0.05; Figure 1e). Intrahypothalamic liraglutide treatment resulted in a significant reduction of inguinal white and interscapular brown adipose tissue compared with subcutaneous liraglutide treatment (*P*<0.01; Figure 1f).

### Central chronic liraglutide treatment reduces brown adipocyte size

**Figure 2:**
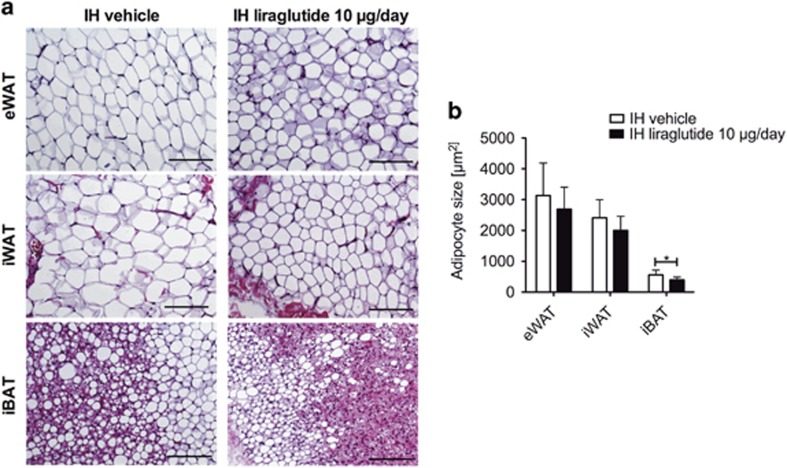
Central intrahypothalamic (IH) administration of liraglutide reduces the size of brown adipocytes. (**a**) Adipocyte size after 28 days of liraglutide administration (IH 10 μg per day). (**b**) Representative hematoxylin and eosin (H&E) stainings of adipose tissues (eWAT, iWAT, iBAT) 28 days after IH liraglutide injection; × 20 magnification, scale bar, 200 μm. Data are given as mean+s.d. of seven to eight animals (*P<0.05).

Brown adipocytes were significantly smaller with chronic intrahypothalamic liraglutide administration compared with the control group (*P*<0.05; Figures 2a and b). We found a trend towards reduced adipocyte size in eWAT and iWAT (Figure 2a).

### Central chronic liraglutide treatment reduces visceral adipose tissue gain

**Figure 3:**
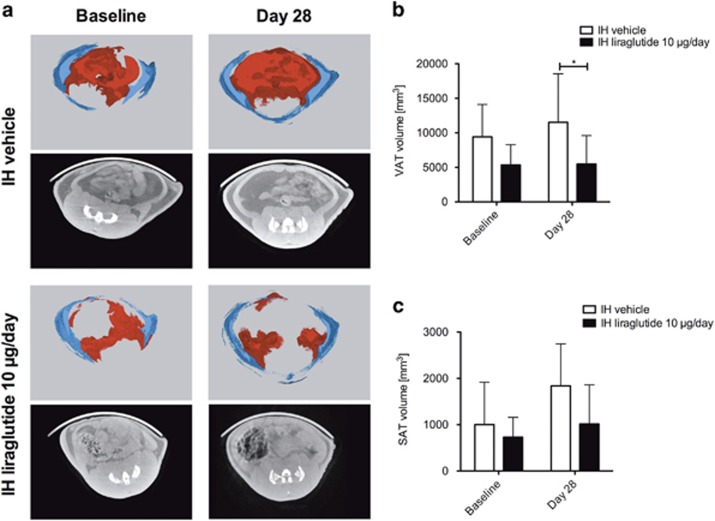
Central intrahypothalamic (IH) administration of liraglutide reduces visceral adipose tissue volume gain. (**a**) μCT calculated visceral adipose tissue (VAT) volume (mm^3^) at baseline (before treatment) and at day 28. (**b**) μCT calculated subcutaneous adipose tissue (SAT) volume (mm^3^) change from baseline to day 28. (**c**) Fully segmented μCT images (xyz) of the abdominal region VAT (red) SAT (blue). Black/white images show transverse axial μCT images of rat abdomen at level L5–S1 intervertebral disk. Data are given as mean+s.d. of seven to eight animals (*P<0.05).

After 28 days of intrahypothalamic liraglutide treatment, we found a significant decrease of visceral adipose tissue compared with the control group (*P*<0.05; Figure 3a). The subcutaneous adipose tissue volume showed a trend towards reduced adipose tissue volume gain by intrahypothalamic liraglutide treatment (Figure 3b) compared with intrahypothalamic vehicle group.

### The liraglutide-induced reduction in body weight and adipose tissue mass is independent of thermogenesis or browning of adipose tissue

**Figure 4:**
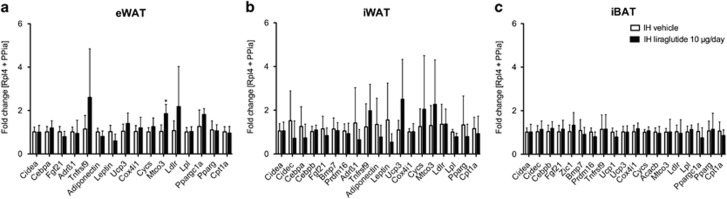
Central intrahypothalamic (IH) administration of liraglutide neither induces browning nor thermogenesis. (**a**) Relative mRNA levels (fold changes) in eWAT in IH liraglutide treatment (28 days) compared with control group (vehicle, aCSF). (**b**) Relative mRNA levels (fold changes) in iWAT of IH 28 days liraglutide treatment compared with control group (vehicle, aCSF). (**c**) Relative mRNA levels (fold changes) in iBAT of IH 28 days liraglutide treatment compared with control group (vehicle, aCSF). *Rpl4* and *PPia* were used as reference genes. Data are given as mean+s.d. of seven to eight animals per group.

Chronic intrahypothalamic liraglutide treatment did not affect expression of brown adipocyte marker (*ucp1*), and expression of browning and adipogenesis markers (*prdm16, cidea, fgf21, tnfrsf9, zic1, bmp7, cebpb, ppargc1a, pparg*) in iWAT, eWAT and iBAT (Figure 4). Mitochondrial cytochrome *c* oxidase subunit 3 (*mtco3*) in eWAT was significantly increased with intrahypothalamic liraglutide administration compared with the control group (*P*<0.05; Figure 4a). We observed a trend towards reduction of leptin and lipoprotein lipase (*lpl*) mRNA levels in eWAT and iWAT with intrahypothalamic liraglutide administration compared with the control group (Figure 4a and b). We observed no changes in gene expression patterns in iBAT (Figure 4c) after intrahypothalamic liraglutide treatment. The subcutaneous liraglutide treatment did not affect expression of thermogenic and browning markers (Supplementary Material).

### Central chronic liraglutide treatment increases circulating plasma thyroxine levels

Chronic intrahypothalamic liraglutide treatment led to a 1.4-fold increase in circulating concentrations of the thyroid hormone thyroxine (T4) on day 28 compared with the control group (*P*<0.05; Table 1). Circulating thyroid-stimulating hormone levels were unaffected by chronic intrahypothalamic and subcutaneous liraglutide treatment compared with control group at treatment day 21 (Table 1). Neither intrahypothalamic nor subcutaneous liraglutide administration affected circulating thyroid-stimulating hormone levels. Neither intrahypothalamic liraglutide nor subcutaneous liraglutide treatment affected any circulating factor of glucose metabolism (insulin, glucose) or fatty acid and lipid metabolism (leptin, NEFA, free glycerol, triglyceride).

### Central chronic liraglutide treatment activates the hypothalamic melanocortin system

**Figure 5:**
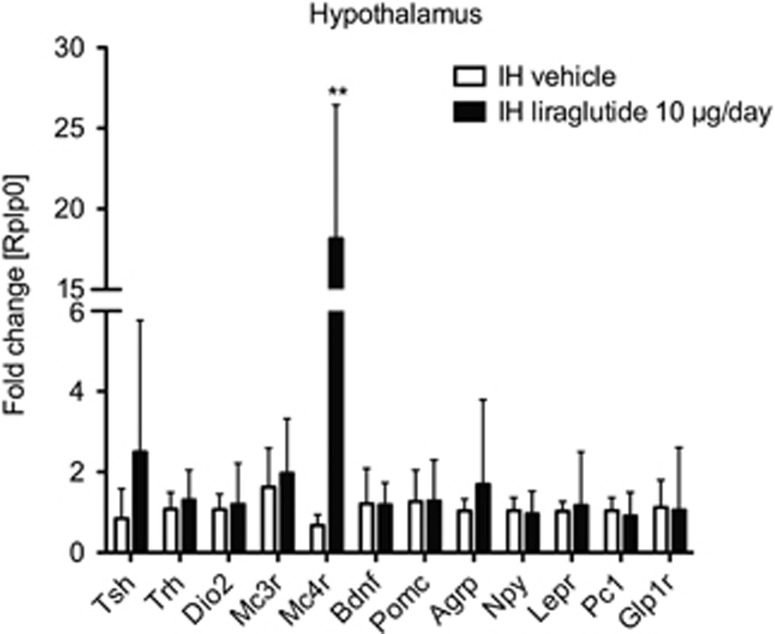
Central intrahypothalamic (IH) liraglutide administration induces hypothalamic MC4R activation. Relative mRNA expression levels (fold changes) of genes involved in pituitary–thyroid axis (*tsh, trh, dio2*) and energy homeostasis (*pomc, bdnf, agrp, npy, lepr, pc1, glp1r, mc3r, mc4r*) in the hypothalamus. *Rplp0* was used as reference gene. Data are given as mean+s.d. of seven to eight animals per group compared with their corresponding control group (**P<0.01).

Chronic intrahypothalamic administration of liraglutide led to an 18-fold induction of the hypothalamic melanocortin 4 receptor gene (*mc4r*) compared with the control group (*P*<0.01; Figure 5) but no further changes were found in any genes regulating food intake and satiety in the hypothalamus (*pomc, bdnf, agrp, npy, lepr, pc1, glp1r*). Expression of appetite regulating peptide *α-msh* was undetectable. Expression of genes of the pituitary–thyroid axis (*tsh, trh, dio2*) was likewise unaffected by intrahypothalamic liraglutide treatment (Figure 5).

## Discussion

This study aimed to identify differential effects of chronic central and peripheral liraglutide treatment. Central application of liraglutide induced a significant body weight loss and overall reduction in body weight gain, which was supported by a significant loss of adipose tissue mass and reduction in visceral adipose tissue gain. Furthermore, chronic intrahypothalamic liraglutide treatment led to a significant activation of hypothalamic *mc4*r gene expression and a significant increase in plasma T4 concentrations.

A significant reduction of body weight gain during the first 16 days of intrahypothalamic liraglutide treatment was observed. The body weight remained lower in the liraglutide-treated group during the last week of treatment. We found that intrahypothalamic liraglutide treatment induced a significant body weight loss from day 9 to day 28 and a significant loss in adipose tissue depots (iWAT, iBAT) after 28 days compared with subcutaneous liraglutide treatment. This is the first study on chronic intrahypothalamic treatment in animal trials but the acute studies (24 h to 6 days) showed similar effects on body weight.^5, 15, 16^ In contrast to acute rat studies with central liraglutide administration,^17, 18^ we showed that chronic (28 days) rather than acute (24 h) intrahypothalamic liraglutide treatment led to a reduction in body weight gain (Supplementary Material). The body weight regain at the end of our study (28 days) could be caused by GLP-1 receptor desensitization (tachyphylaxis) on continuous stimulation^19^ supported by the unchanged GLP-1R expression in the hypothalamus in our study after chronic intrahypothalamic liraglutide administration. Other physiological mechanisms such as reduced energy expenditure, increased appetite seen as an increase in ghrelin,^20^ reduced satiety or a reduction in plasma leptin could be further explanations for the observed body weight regain.^21, 22, 23, 24^ As we investigated the liraglutide effects in a healthy lean animal model without leptin resistance, an observed trend towards reduced plasma leptin levels after chronic intrahypothalamic treatment could be responsible for the body weight regain, as this reduction has been suggested to increase caloric intake.^21, 24^ Thus, the body weight regain seems to be a natural protective counter-regulatory physiological response after weight loss, also seen in humans.^3^

In humans, 3 mg liraglutide causes a moderate weight loss of 6–8 kg in obese individuals with slight weight regain.^3, 4^ Our animal study did not result in any reduction in body weight gain after subcutaneous chronic administration of liraglutide while similar subcutaneous liraglutide treatment has led to anorectic effects and body weight loss in diet-induced obese rats on high-fat diet.^8^ A more pronounced anorectic effect can be expected in such high-fat diet studies^25, 26^ compared with our study design with lean rats on normal diet with the primary aim to test liraglutide effects in a healthy animal model.

Furthermore, the anorectic effect and body weight loss are affected by the liraglutide dosing regimen,^3, 27, 28^ the maximum dose of liraglutide used as obesity treatment is 3 mg, whereas 1.2 mg or 1.8 mg are used for the treatment of type 2 diabetes. Such high dosing is necessary to decrease the caloric intake and reduce body weight compared with lower dosing, which is already sufficient to increase insulin secretion and lower blood glucose levels. Considering the measured liraglutide concentration in plasma on day 28 (2000 pmol l^−1^±975), we assume that the continuous subcutaneous dosing of liraglutide in our study was not effective enough to stimulate the hypothalamic GLP-1 receptor to trigger body weight loss compared with a more effective bolus administration in other studies.^8, 28^

Our combined results on body weight gain and adipose tissue reduction indicate that liraglutide exerts its body weight-reducing effects more potently via central rather than peripheral mechanisms.

A recent 24 h study associated the acute administration of liraglutide with increased thermogenesis in brown adipose tissue and browning of white adipose tissue through the AMP-activated protein kinase pathway in the ventromedial hypothalamus independent of food intake.^5^ We found no significant changes in gene expression levels regarding the brown adipocyte marker *ucp1*, transcription factors for browning/beiging (*prdm16, ppargc1a*), browning/beiging-enriched markers (*cidea, cidec, tnfrsf9, zic1, adrβ1*) and browning/beiging activators (*fgf21, bmp7*).^29, 30, 31, 32, 33^ Our study does not support the involvement of increased thermogenic or browning capability in liraglutide-induced weight loss but this could be due to differences in the delivery site.^5^ Several animal and human studies have attributed the weight-reducing effects mediated by GLP-1R agonists to caloric intake rather than to increased energy expenditure^6, 8, 34, 35^ but our study was not designed to address this difference in more detail.

Similar to the reduction in body weight gain, the chronic intrahypothalamic liraglutide treatment in our study led to a sustained reduction of eWAT and iWAT mass as well as a reduced gain in visceral adipose tissue volume, but we found no such effect after subcutaneous liraglutide administration. White adipocytes showed a trend towards reduced size in eWAT and iWAT after intrahypothalamic liraglutide administration. IBAT mass was unaffected, whereas iBAT adipocytes were significantly reduced in size after chronic intrahypothalamic liraglutide treatment. A previous study using acute liraglutide treatment in diet-induced obese mice attributed a 14-day fat mass reduction to central GLP-1 receptor signaling,^6^ which supports our assumption that the reduction in visceral adipose tissue is mainly mediated by hypothalamic neural mechanisms as indicated by the clear difference between the effects of intrahypothalamic and subcutaneous liraglutide administration found in our study.

Hormonal parameters for glucose metabolism and fatty acid metabolism were largely unaffected by chronic intrahypothalamic liraglutide treatment except a significant increase in circulating T4 concentrations. In humans, weight loss and maintenance of body weight have been associated with increased peripheral conversion of T4 to the bioactive enantiomer reverse T3^23,36^ but we found no changes in the expression of type II iodothyronine deiodinase (DIO2) in iBAT (data not shown), which is required for the intracellular conversion of T4 to T3.^37^

We also tested the effect of chronic intrahypothalamic liraglutide administration on neuronal centers known to regulate caloric intake and energy homeostasis. In contrast to other studies, we observed no change in the hypothalamic mRNA content of orexigenic (*agrp*) or anorexigenic (*pomc*) neurons, but we found a significant 18-fold induction of *mc4r* mRNA in the hypothalamus after 28 days of intrahypothalamic liraglutide administration. These results indicate an alternative pathway for liraglutide to activate the anorectic MC4R was besides the previously described POMC/CART produced melanocyte-stimulating hormone α-MSH signaling in the hypothalamus.^38, 39, 40^ To our knowledge, we are first to show that continuous, chronic liraglutide administration in the hypothalamus leads to a significant activation of the MC4R. Recently, a 5-day combination therapy including the MC4R agonist RM-493 and liraglutide in diet-induced obese mice showed improved body weight and fat mass reduction compared with monotherapy.^41^ There is also evidence that the melanocortin signaling is involved in the reward system,^42^ which was previously reported as high-fat fed or candy-fed rats prefer low-fat or chow diet after the administration of MC4R agonists (MTII).^43, 44^

We conclude that continuous chronic intrahypothalamic liraglutide treatment rather than peripheral administration leads to an improvement in terms of reduced body weight and adipose tissue weight and unchanged metabolic regulators. The activation of the anorectic MC4R mechanism shows that liraglutide is a potent regulator of energy homeostasis and that there is an alternative pathway to stimulate the melanocortin system other than POMC/α-MSH. Identification of novel anorectic targets in the hypothalamus triggering the melanocortin system and its downstream system can help to develop safe low-dose combination therapies to reduce side effects and improve the chronic response and metabolic control.

Follow-up studies will help to disclose new targets linking the melanocortin and thyroid systems responsible for the chronic effects of liraglutide and consequently new therapies for obesity treatment.

## Figures and Tables

**Table 1:** Central IH administration of liraglutide increases circulating T4 concentration

	*IH vehicle*	*IH liraglutide* *(10 μg per day)*	*SC vehicle*	*SC liraglutide* *(200 μg kg^−1^ per day)*
FG (μmol ml^−1^)	13.21±2.94	13.43±2.73	11.98±1.54	11.39±1.71
NEFA (μmol ml^−1^)	14.67±4.14	15.54±5.4	15.85±5.0	17.76±5.77
TAG (μmol l^−1^)	0.08±0.02	0.08±0.03	0.10±0.03	0.10±0.04
Leptin (ng ml^−1^)	25.20±14.0	14.96±13.54	8.20±5.74	8.18±5.48
Insulin (ng ml^−1^)	1.71±0.61	1.23±0.45	1.24±0.57	1.07±0.12
Glucose (mmol l^−1^)	7.04±0.33	7.29±0.69	7.90±0.63	6.56±0.89
T3 (ng dl^−1^)	24.35±5.6	25.95±7.3	24.99±8.6	24.34±7.8
T4 (ng dl^−1^)	57.78±8.2	80.61±23.3*	61.0±12.6	58.42±14.8
TSH (ng ml^−1^) (treatment day 21)	*1.20±0.30^a^*	*1.12±0.35^a^*	*1.75±1.20^a^*	*1.20±0.31^a^*

Abbreviations: FG, free glycerol; NEFA, non-esterified fatty acids; T3, triiodothyronine; T4, thyroxine; TAG, triglyceride; TSH, thyroid-stimulating hormone.

a TSH levels are only measured for day 21 due to lack of samples.

Circulating plasma levels of markers for glucose metabolism (glucose, insulin), lipid metabolism (leptin, NEFA, TAG, FG) and thyroid hormones (T3, T4) at day 28 after chronic intrahypothalamic (IH; 10 μg per day) and subcutaneous (SC; 200 μg kg^−1^ per day) liraglutide treatment. Data are given as mean±s.d. of seven to eight animals per group (*P<0.05). Italic values are indicate a different time point of TSH measurements compared to the other plasma hormone levels.
