# Supplementary material for: Chronic intrahypothalamic rather than subcutaneous liraglutide treatment reduces body weight gain and stimulates the melanocortin receptor system
Source: Int J Obes (Lond). 2017 May 16;41(8):1263–70. doi: 10.1038/ijo.2017.98 (PMC5550563; doi:10.1038/ijo.2017.98)
Supplement: Supplementary Information [file ijo201798x1.docx]

## SYBR or TaqMAN primer sequences used for RT-PCR analysis. The primers were used to analyze thermogenic and browning gene expression patterns in the epididymal, inguinal white and interscapular brown adipose tissue and for assessment of mRNA levels of appetite regulators in the hypothalamus.

**Table 1-** Primer sequences designed by using NCBI Blast (Standard Nucleotide BLAST); all self-designed primers were tested for performance by agarose gel electrophoresis.

| Oligo name | 5’3’ Primer |
| --- | --- |
| Rplp0 | FW *AAGCAAAGGAAGAGTCGGAGG*  RV *TGCAAATGGATGCGAGCAAG* |
| B2m | FW *GTGCTTAGCAGCCTAGCAGT*  RV *GATGAAAACCGCACACAGGC* |
| Mc4r | FW *CGGGTCAGAAACCATCGTCA*  RV *TGCAAATGGATGCGAGCAAG* |
| Mc3r | FW *TTATCCGACGCTGCCTAACC*  RV *CATCAGACTGACGATGCCCA* |
| Pc1 | FW *AGTAAAGCAACCCAGAGCCAG*  RV *CTTTGCTTCATGGCTCGCAC* |
| Pomc | FW *GACCTCACCACGGAAAGCAA*  RV *TGACCCATGACGTACTTCCG* |
| Agrp | FW *AGGAAGTAGTCACGTGTGGG*  RV *GGACACAGCTCAGCAACATT* |
| aMsh | FW *GCGATCTAGAACCCGACTGT*  RV *GAGGCTCACATCTGCATGGT* |
| Socs3 | FW *AGAACCTACGCATCCAGTGC*  RV *GGTTCCGTCGGTGGTAAAGA* |
| Lepr | FW *CCTGCTGGAGTCCCAAACAA*  RV *GCGGAGCAGTTTTGACCTTG* |
| Trh | FW *ACTCTTCAGCTCAGCATCTTGG*  RV *AGGGTGAAGATCAAAGCCAGA* |
| Dio2 | FW *AAGTGTCCCCTTCGGTTTCC*  RV *ATGGTACGCGCACATTACCT* |
| Tsh | FW *GAAATACCGGGATGCCCACA*  RV *TCTGTGGCTTGGTGCAGTAG* |
| Glp1r | FW *ACAGGTCTCTTCTGCAACCG*  RV *ATGCCCTTGGAGCACACTAC* |

| Oligo name | 5’3’ Primer – TaqMan probes  Commercially primers provided by Life Technologies |
| --- | --- |
| Ppia | Rn00690933_m1 |
| Rpl4 | Rn00821091_g1 |
| Hprt | Rn01527840_m1 |
| Ucp1 | Rn00562126_m1 |
| Ucp3 | Rn00565874_m1 |
| Cidea | Rn04181355_m1 |
| Cidec | Rn01421167_m1 |
| Fgf21 | Rn00590706_m1 |
| Cebpa | Rn00560963_s1 |
| Cebpb | Rn00824635_s1 |
| Adrß1 | Rn00824536_s1 |
| Ppargc1α | Rn00580241_m1 |
| Pparγ | Rn00440945_m1 |
| Adiponectin | Rn00595250_m1 |
| Leptin | Rn00565158_m1 |
| Prdm16 | Rn01516224_m1 |
| Zic1 | Rn00575376_m1 |
| Bmp7 | Rn01528889_m1 |
| Mtco3 | Rn03296820_s1 |
| Ldlr | Rn00598442_m1 |
| Lpl | Rn00561482_m1 |
| Cox4i1 | Rn00665001_g1 |
| Cpt1a | Rn00580702_m1 |
| Cycs | Rn01410227_g1 |
| Acacb | Rn00588290_m1 |

## Supplementary methods

**Verification of cannula placement**

The cannula was inserted into the rat brain directly to the border of the hypothalamus
(-1.7/0.6/7.6 mm) according to the rat brain coordinates (Paxinos & Watson Rat Brain Atlas). The cannula was rinsed with 2 µL of 0.9% NaCl (Fresenius Kabi, Graz, Austria) and a volume of 3 µL of sodium fluorescein (Naf) (7.5 mg/mL diluted in 0.9% NaCl) was injected via the cannula to the hypothalamus (flow rate 0.1 µL/min). Next the rat was decapitated, the brain was extracted, and the hypothalamus was dissected. The frontal cortex was dissected as well and used as negative control. The tissue was homogenized and sterile water (Aqua bidest; Fresenius Kabi, Graz, Austria) was added (w/v). The homogenized tissue was left in the fridge at 4°C over night to allow Naf to diffuse to the aqueous phase. The following day, the supernatant was collected and analysed by using the Microplate Reader Synergy HT (Biotek, Vienna, Austria) (emission wavelength of 485 nm) at Joanneum Research HEALTH (Graz, Austria).

In addition we implanted the brain cannula (1.7/0.6/7.6 mm), waited for 30 minutes and perfused the rat with 4% PFA for rapid fixation of the brain. The brain was fixed in 4% PFA over night at 4°C, washed three times in PBS and incubated in 20% sucrose in PBS solution over night at 4°C. Brains are then stored at -20°C in TissueTek wells embedded in OCT solution till Cryocutting and H&E staining.

## Supplementary results


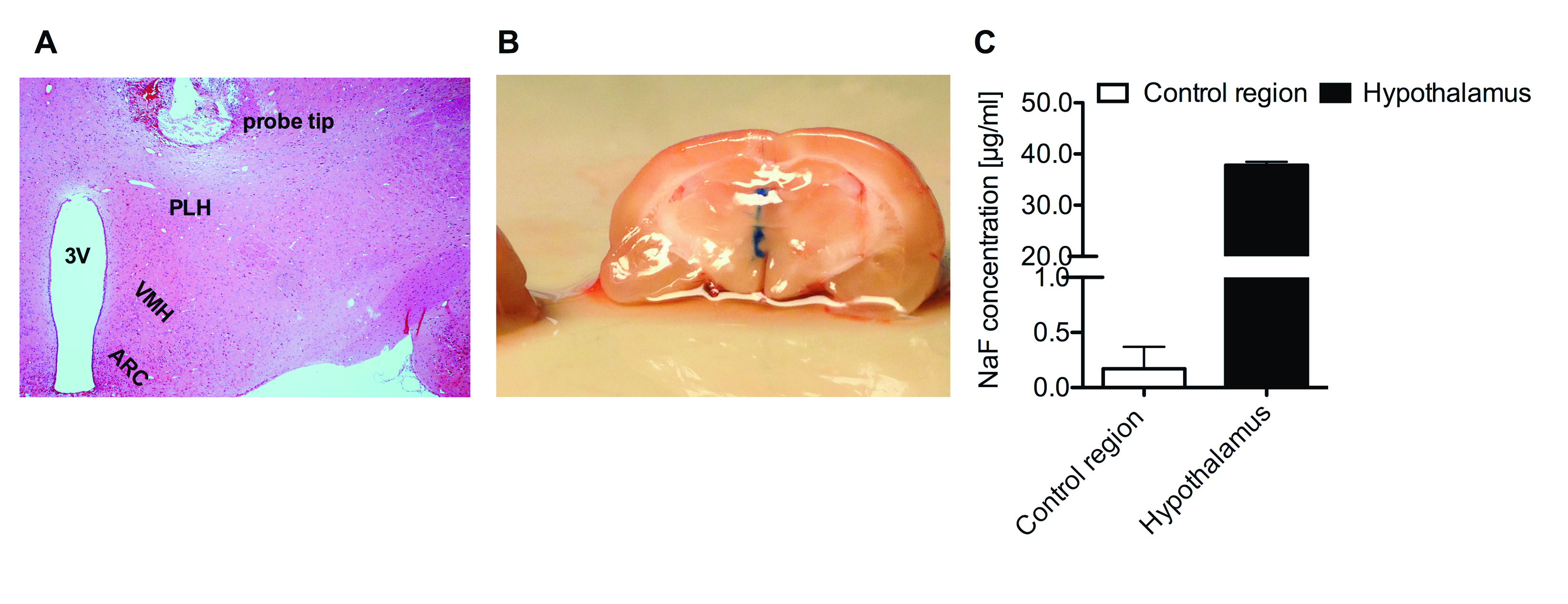


**Figure 1 – Verified cannula placement quantitatively and qualitatively**. (**A**) H&E staining of a 5-µm thick section of the rat hypothalamus with the indicated correct placement of the implanted cannula probe tip (-1.7/0.6/7.6 mm); PLH (peduncular part of the lateral hypothalamus), VMH (ventromedial hypothalamus), ARC (arcuate nucleus), 3V (third ventricle); (**B**) Stained (blue line) cannula channel and probe tip in the rat brain by using evans blue as marker. (**C**) Qualitative assessment of intrahypothalamic Naf administration via the used brain cannula.

Chronic SC liraglutide treatment for 28 days did not affect the gene expression of thermogenic and browning markers in adipose tissue depots (eWAT, iWAT, iBAT).


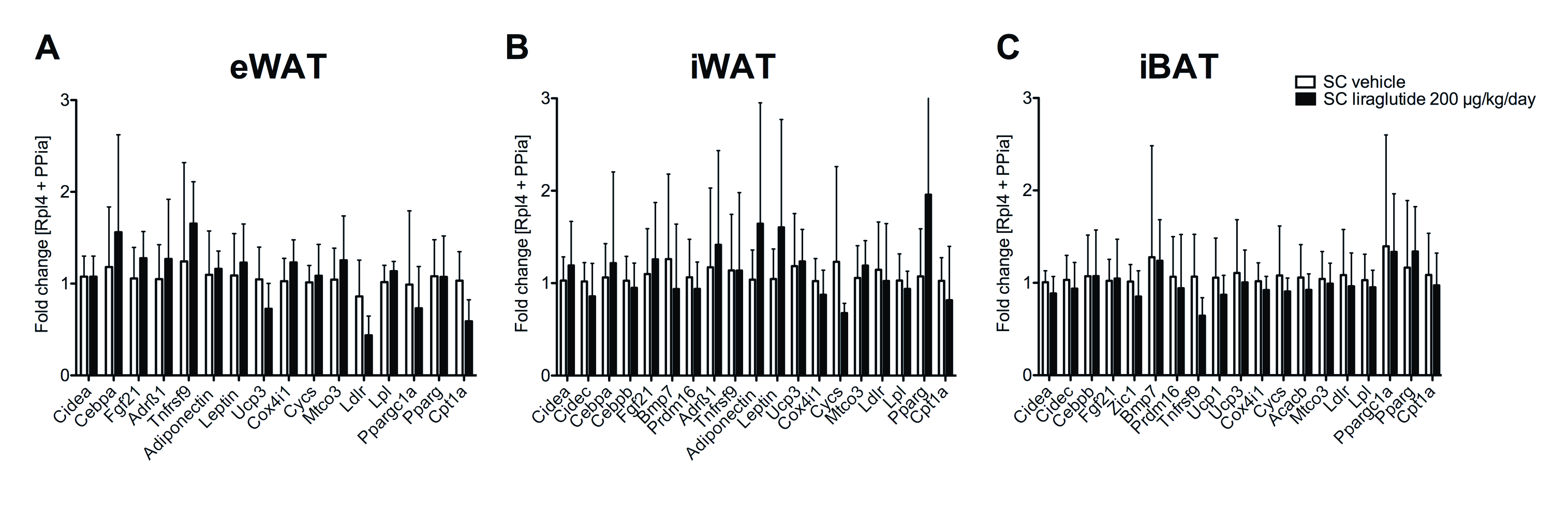


**Figure 2 – Peripheral SC administration of liraglutide neither induces browning nor thermogenesis.** (**A**) Relative mRNA levels (fold changes) in eWAT in SC liraglutide treatment (28 days) compared to control group (SC vehicle, 0.9% NaCl). (**B**) Relative mRNA levels (fold changes) in iWAT in SC liraglutide treatment (28 days) compared to control group (SC vehicle, 0.9% NaCl). (**C**) Relative mRNA levels (fold changes) in iBAT in SC liraglutide treatment (28 days) compared to control group (SC vehicle, 0.9% NaCl). *Rpl4* and *PPia* were used as reference genes. Data are given in mean + SD of 7-8 animals per group.

Here we investigated the acute effects of intrahpothalamic (IH) liraglutide (10µg) effects on body weight, adiopose tissue weights, expression of thermogenic and browning factors in the white and brown adipose tissue, and the acute effect on the expression of anorexigenic and orexigenic appetite regulators in the hypothalamus. Our acute study resulted in unchanged body weight and adipose tissue weights after IH liraglutide treatment compared to control group (IH vehicle). We observed unchanged gene expression patterns of thermogenic and browning factors in the adipose tissue and the expression of appetite regulators in the hypothalamus was unaffected too.


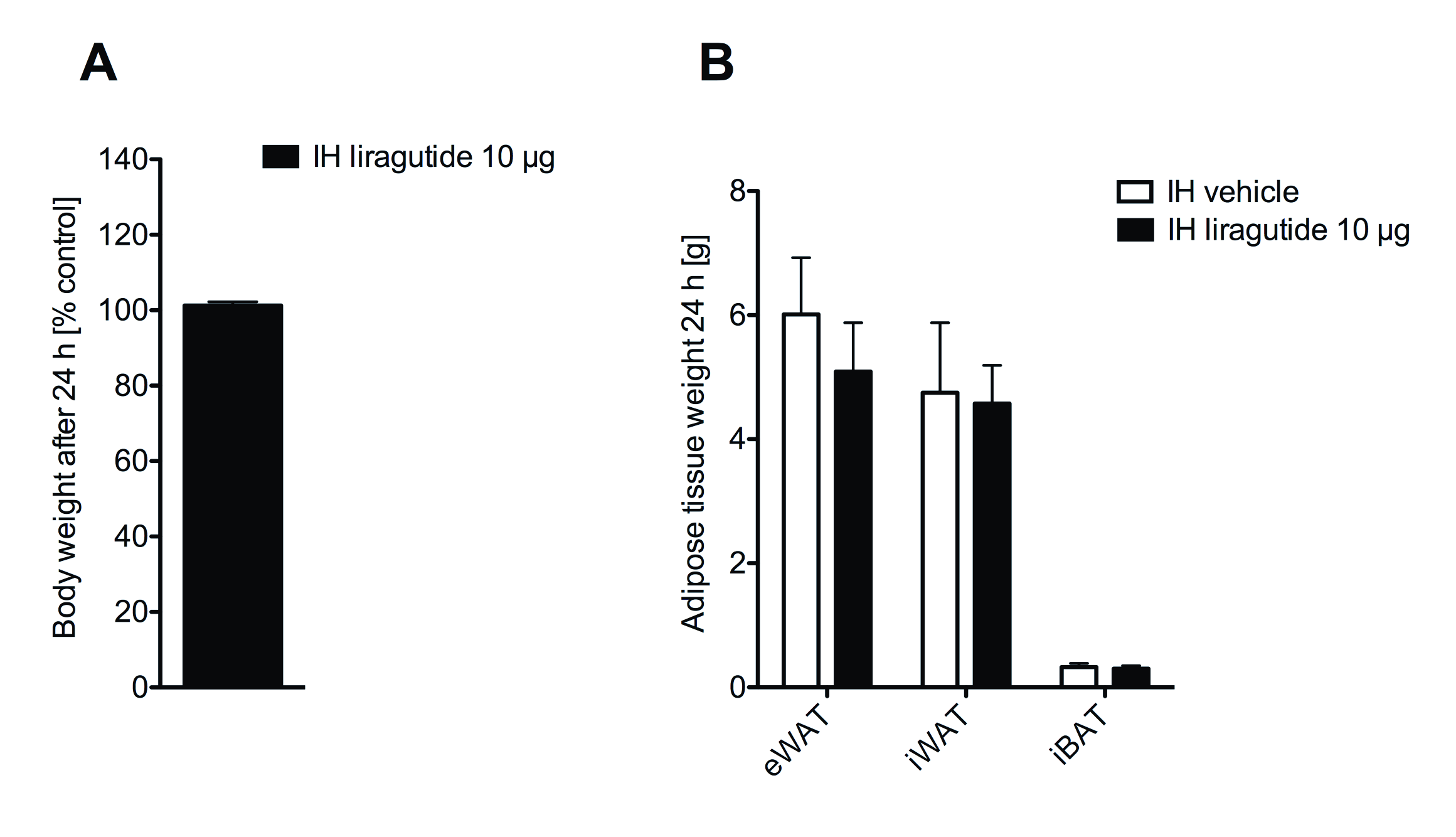


**Figure 3 – Acute intrahypothalamic (IH) liraglutide treatment does not affect body weight or adipose tissue weight.** (**A**) Body weight after IH liraglutide treatment for 24h compared to IH vehicle (control group). (**B**) Adipose tissue weights of epididymal, inguinal white and interscapular brown adipose tissue (eWAT, iWAT, iBAT) after IH liraglutide treatment for 24h compared to IH vehicle. Results are indicated as mean + SD.


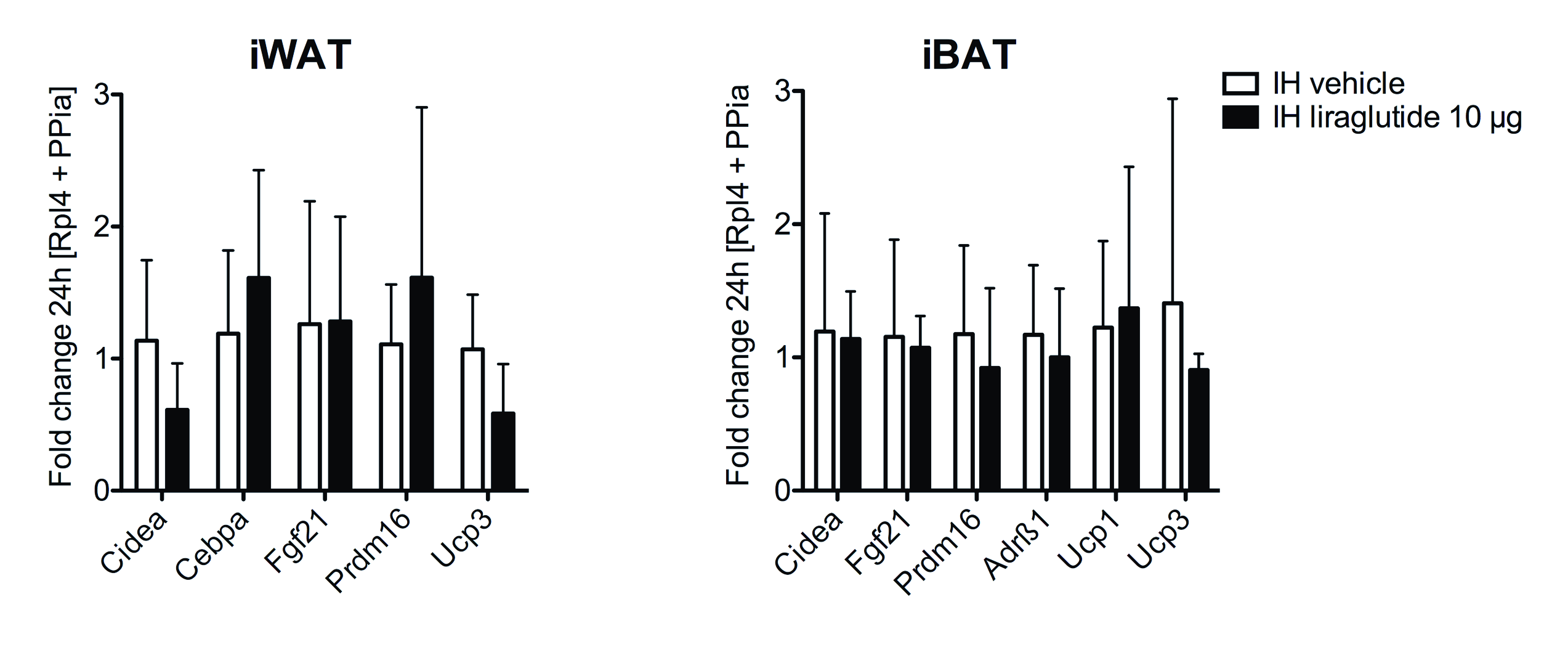


**Figure 4 – Acute liraglutide administration does not affect the expression of thermogenic and browning factors in adipose tissue**. 24 hours intrahypothalamic (IH) liraglutide treatment shows no effect on thermogenic (*ucp3, ucp1,adrß1*) and browning (*cidea, cebpa, fgf21, prdm16*) mRNA content in inguinal (iWAT) white and brown (iBAT) adipose tissue. *Rpl4* and *Ppia* were used as reference genes. Results are indicated as mean + SD.


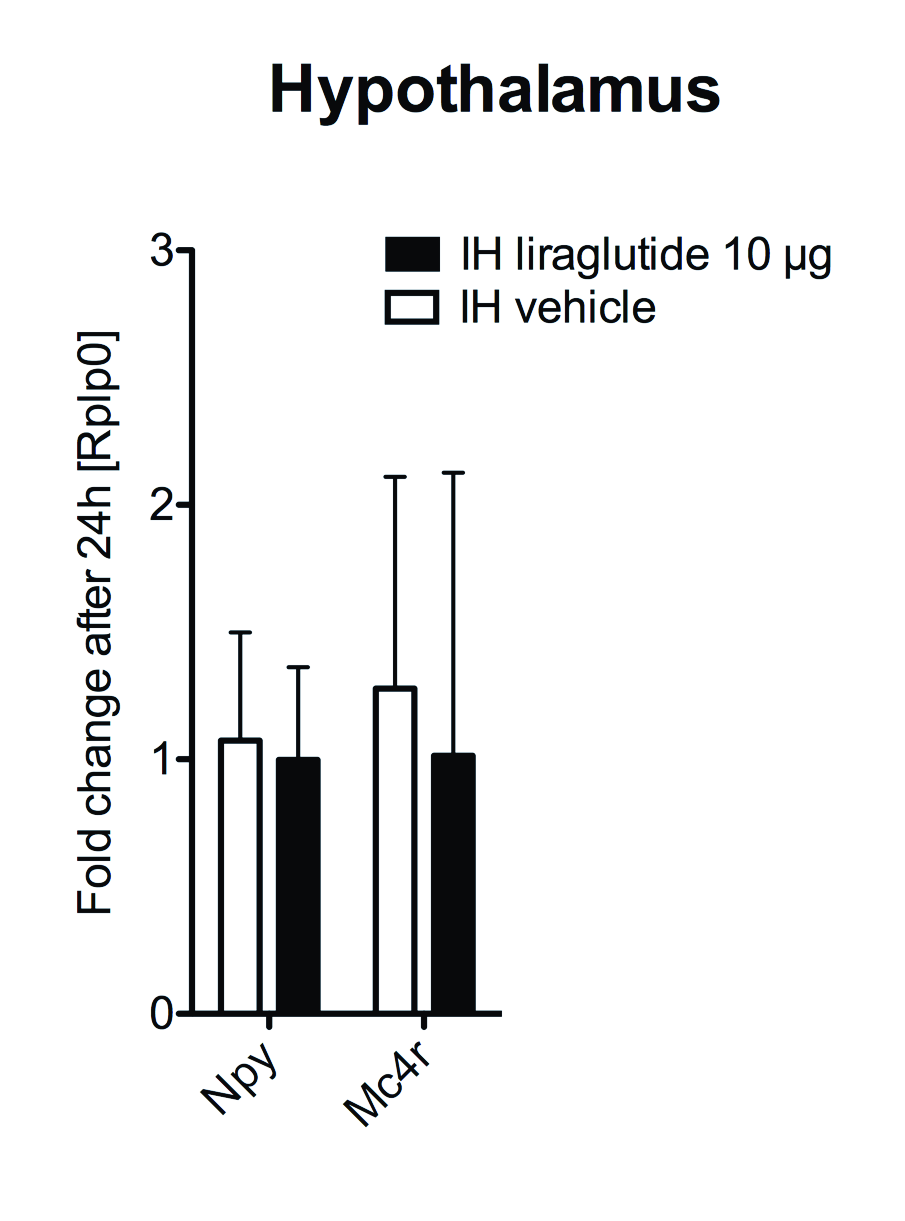


**Figure 5 – Acute intrahypothalamic liraglutide treatment does not change hypothalamic appetite regulators**. 24 hours intrahypothalamic liraglutide treatment (10 µg) does not reduce orexigenic *npy* mRNA content or stimulate anorexigenic *mc4r* mRNA content in the hypothalamus. *Rplp0* was used as reference gene. Results are indicated as mean + SD.
